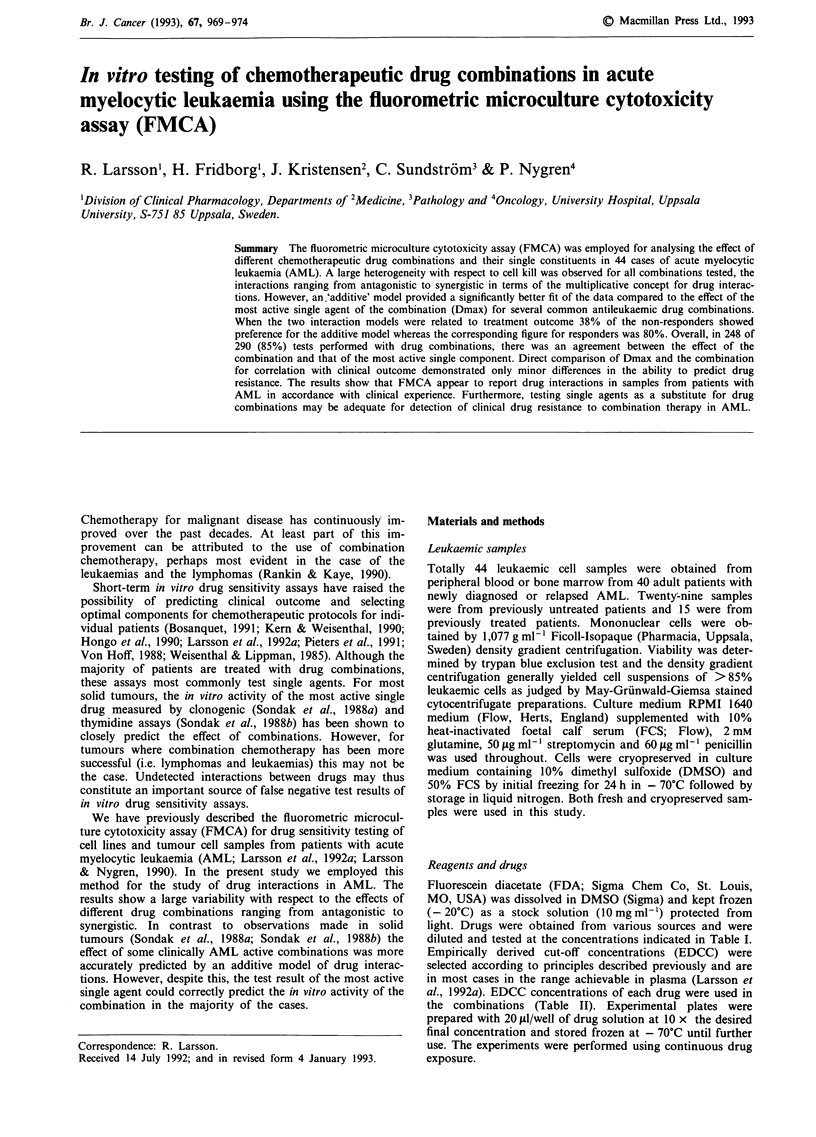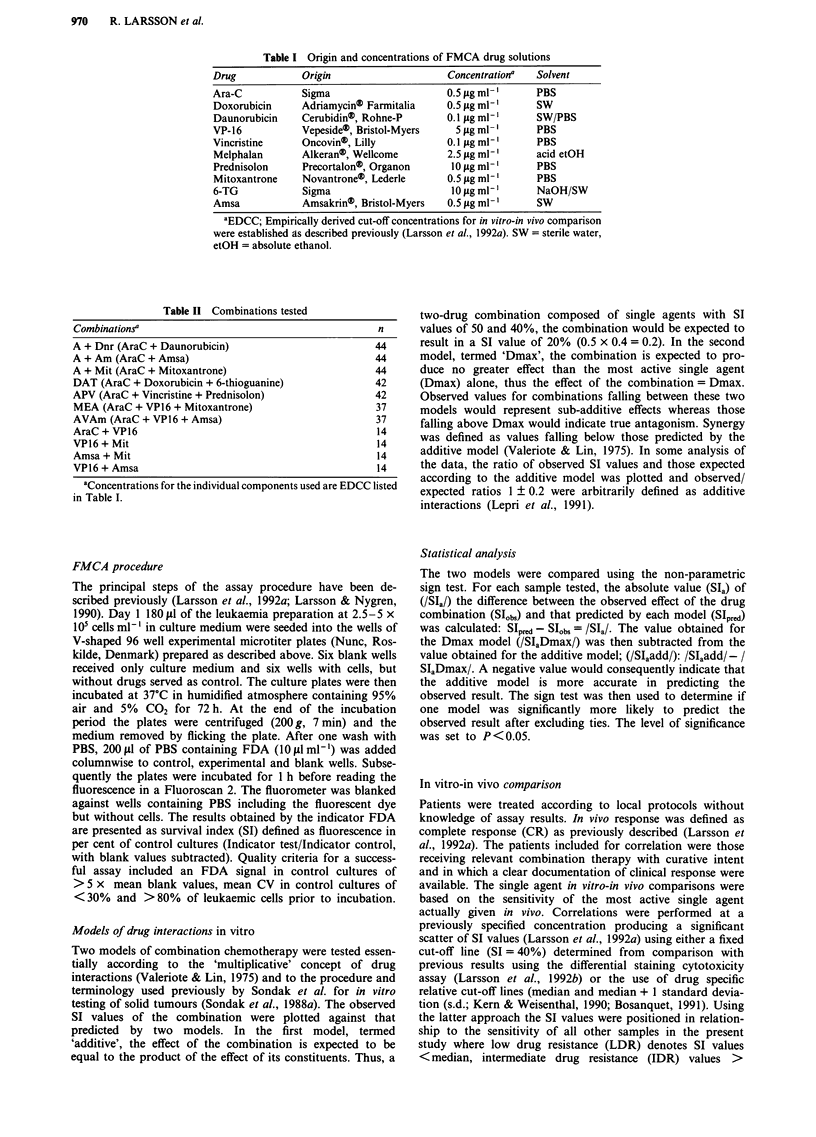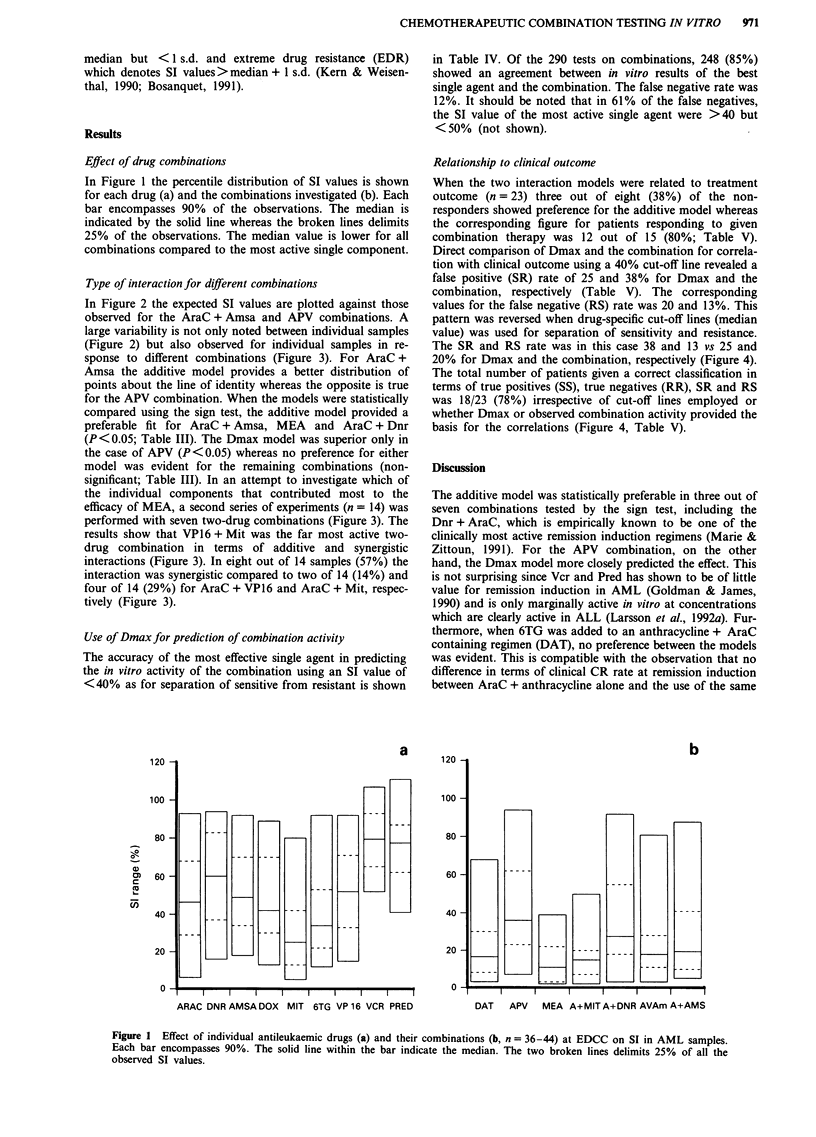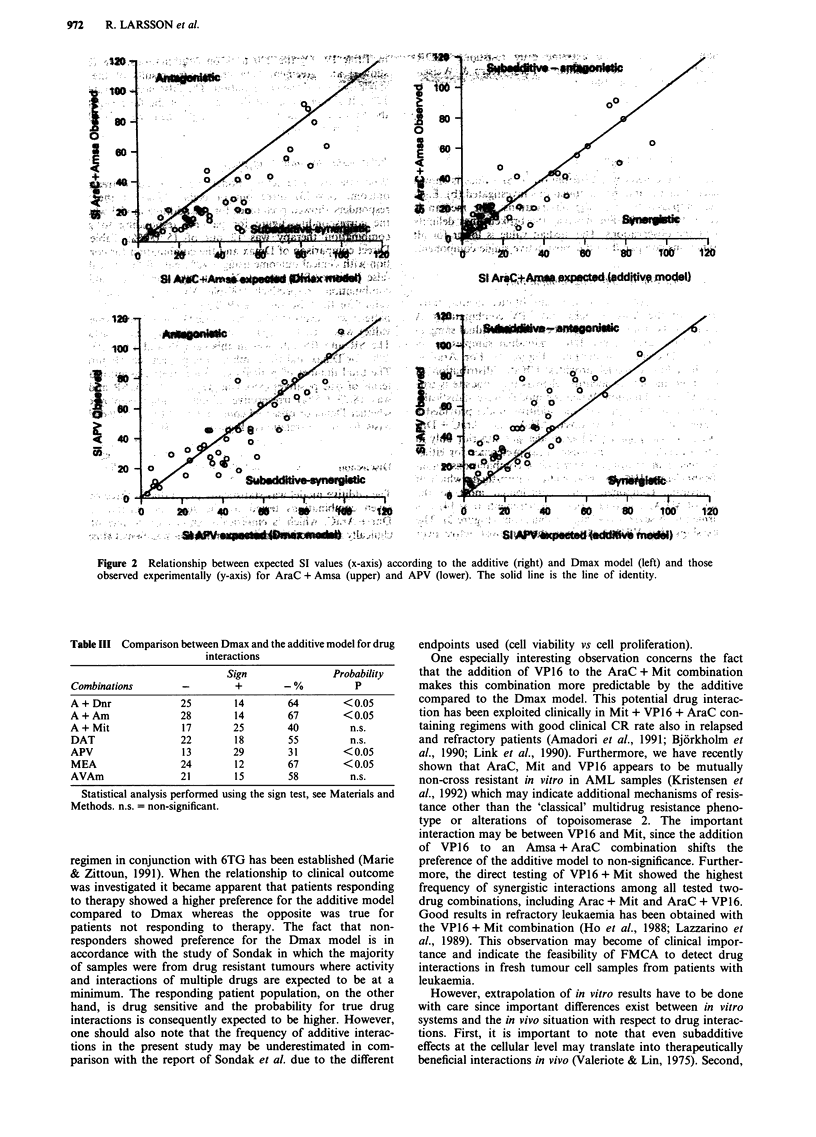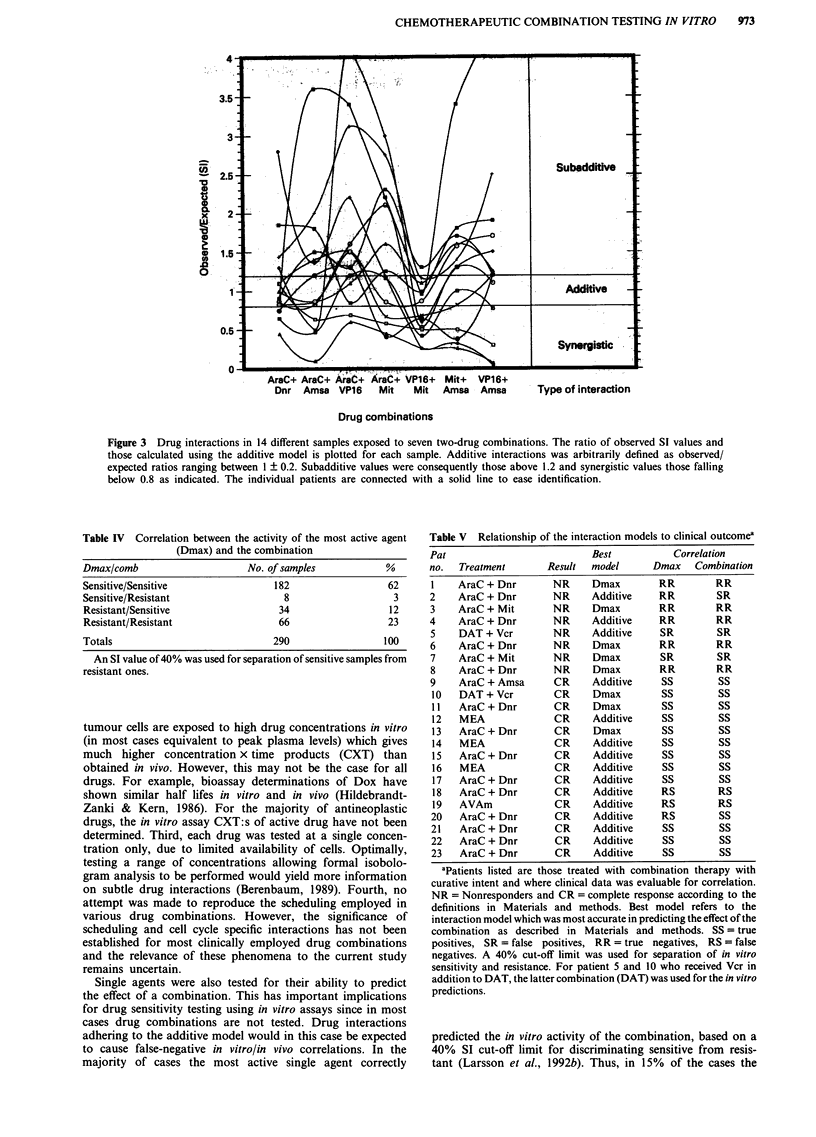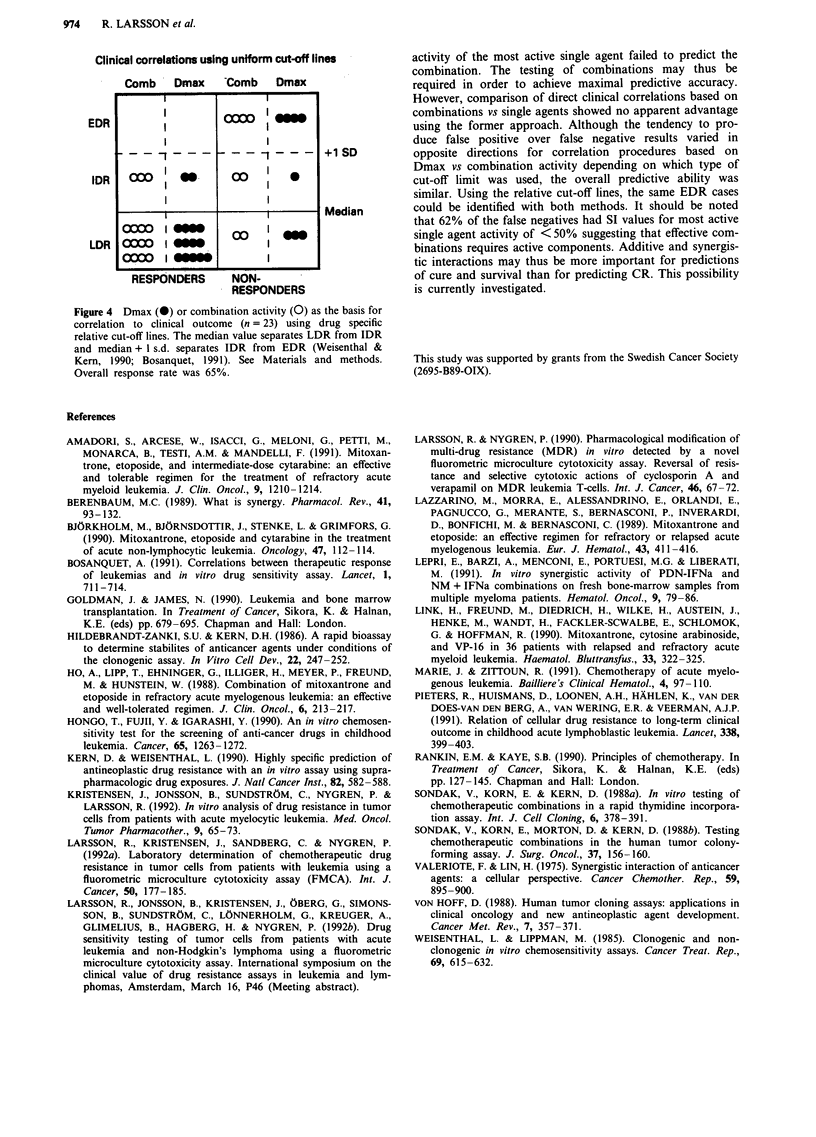# In vitro testing of chemotherapeutic drug combinations in acute myelocytic leukaemia using the fluorometric microculture cytotoxicity assay (FMCA).

**DOI:** 10.1038/bjc.1993.178

**Published:** 1993-05

**Authors:** R. Larsson, H. Fridborg, J. Kristensen, C. Sundström, P. Nygren

**Affiliations:** Division of Clinical Pharmacology, University Hospital, Uppsala University, Sweden.

## Abstract

The fluorometric microculture cytotoxicity assay (FMCA) was employed for analysing the effect of different chemotherapeutic drug combinations and their single constituents in 44 cases of acute myelocytic leukaemia (AML). A large heterogeneity with respect to cell kill was observed for all combinations tested, the interactions ranging from antagonistic to synergistic in terms of the multiplicative concept for drug interactions. However, an 'additive' model provided a significantly better fit of the data compared to the effect of the most active single agent of the combination (Dmax) for several common antileukaemic drug combinations. When the two interaction models were related to treatment outcome 38% of the non-responders showed preference for the additive model whereas the corresponding figure for responders was 80%. Overall, in 248 of 290 (85%) tests performed with drug combinations, there was an agreement between the effect of the combination and that of the most active single component. Direct comparison of Dmax and the combination for correlation with clinical outcome demonstrated only minor differences in the ability to predict drug resistance. The results show that FMCA appear to report drug interactions in samples from patients with AML in accordance with clinical experience. Furthermore, testing single agents as a substitute for drug combinations may be adequate for detection of clinical drug resistance to combination therapy in AML.


 © Macmillan Press Ltd., 1993

### *In vitro* testing of chemotherapeutic drug combinations in acute myelocytic leukaemia using the fluorometric microculture cytotoxicity assay (FMCA)

R. Larsson[1], H. Fridborg[1], J. Kristensen[2], C. Sundström[3] & P. Nygren[4]

[1]*Division of Clinical Pharmacology, Departments of* [2]*Medicine,* [3]*Pathology and* [4]*Oncology, University Hospital, Uppsala University, S-751 85 Uppsala, Sweden.*

**Summary** The fluorometric microculture cytotoxicity assay (FMCA) was employed for analysing the effect of different chemotherapeutic drug combinations and their single constituents in 44 cases of acute myelocytic leukaemia (AML). A large heterogeneity with respect to cell kill was observed for all combinations tested, the interactions ranging from antagonistic to synergistic in terms of the multiplicative concept for drug interactions. However, an 'additive' model provided a significantly better fit of the data compared to the effect of the most active single agent of the combination (Dmax) for several common antileukaemic drug combinations. When the two interaction models were related to treatment outcome 38% of the non-responders showed preference for the additive model whereas the corresponding figure for responders was 80%. Overall, in 248 of 290 (85%) tests performed with drug combinations, there was an agreement between the effect of the combination and that of the most active single component. Direct comparison of Dmax and the combination for correlation with clinical outcome demonstrated only minor differences in the ability to predict drug resistance. The results show that FMCA appear to report drug interactions in samples from patients with AML in accordance with clinical experience. Furthermore, testing single agents as a substitute for drug combinations may be adequate for detection of clinical drug resistance to combination therapy in AML.

Chemotherapy for malignant disease has continuously improved over the past decades. At least part of this improvement can be attributed to the use of combination chemotherapy, perhaps most evident in the case of the leukaemias and the lymphomas (Rankin & Kaye, 1990).

Short-term *in vitro* drug sensitivity assays have raised the possibility of predicting clinical outcome and selecting optimal components for chemotherapeutic protocols for individual patients (Bosanquet, 1991; Kern & Weisenthal, 1990; Hongo *et al.*, 1990; Larsson *et al.*, 1992*a*; Pieters *et al.*, 1991; Von Hoff, 1988; Weisenthal & Lippman, 1985). Although the majority of patients are treated with drug combinations, these assays most commonly test single agents. For most solid tumours, the *in vitro* activity of the most active single drug measured by clonogenic (Sondak *et al.*, 1988*a*) and thymidine assays (Sondak *et al.*, 1988*b*) has been shown to closely predict the effect of combinations. However, for tumours where combination chemotherapy has been more successful (i.e. lymphomas and leukaemias) this may not be the case. Undetected interactions between drugs may thus constitute an important source of false negative test results of *in vitro* drug sensitivity assays.

We have previously described the fluorometric microculture cytotoxicity assay (FMCA) for drug sensitivity testing of cell lines and tumour cell samples from patients with acute myelocytic leukaemia (AML; Larsson *et al.*, 1992*a*; Larsson & Nygren, 1990). In the present study we employed this method for the study of drug interactions in AML. The results show a large variability with respect to the effects of different drug combinations ranging from antagonistic to synergistic. In contrast to observations made in solid tumours (Sondak *et al.*, 1988*a*; Sondak *et al.*, 1988*b*) the effect of some clinically AML active combinations was more accurately predicted by an additive model of drug interactions. However, despite this, the test result of the most active single agent could correctly predict the *in vitro* activity of the combination in the majority of the cases.

Correspondence: R. Larsson.
Received 14 July 1992; and in revised form 4 January 1993.

### Materials and methods

### *Leukaemic samples*

Totally 44 leukaemic cell samples were obtained from peripheral blood or bone marrow from 40 adult patients with newly diagnosed or relapsed AML. Twenty-nine samples were from previously untreated patients and 15 were from previously treated patients. Mononuclear cells were obtained by 1,077 g ml$^{-1}$ Ficoll-Isopaque (Pharmacia, Uppsala, Sweden) density gradient centrifugation. Viability was determined by trypan blue exclusion test and the density gradient centrifugation generally yielded cell suspensions of >85% leukaemic cells as judged by May-Grünwald-Giemsa stained cytocentrifugate preparations. Culture medium RPMI 1640 medium (Flow, Herts, England) supplemented with 10% heat-inactivated foetal calf serum (FCS; Flow), 2 mM glutamine, 50 μg ml$^{-1}$ streptomycin and 60 μg ml$^{-1}$ penicillin was used throughout. Cells were cryopreserved in culture medium containing 10% dimethyl sulfoxide (DMSO) and 50% FCS by initial freezing for 24 h in − 70°C followed by storage in liquid nitrogen. Both fresh and cryopreserved samples were used in this study.

### *Reagents and drugs*

Fluorescein diacetate (FDA; Sigma Chem Co, St. Louis, MO, USA) was dissolved in DMSO (Sigma) and kept frozen (− 20°C) as a stock solution (10 mg ml$^{-1}$) protected from light. Drugs were obtained from various sources and were diluted and tested at the concentrations indicated in Table I. Empirically derived cut-off concentrations (EDCC) were selected according to principles described previously and are in most cases in the range achievable in plasma (Larsson *et al.*, 1992*a*). EDCC concentrations of each drug were used in the combinations (Table II). Experimental plates were prepared with 20 μl/well of drug solution at 10 × the desired final concentration and stored frozen at − 70°C until further use. The experiments were performed using continuous drug exposure.

**Table I** Origin and concentrations of FMCA drug solutions

| *Drug* | *Origin* | *Concentration*[a] | *Solvent* |
|---|---|---|---|
| Ara-C | Sigma | 0.5 μg ml$^{-1}$ | PBS |
| Doxorubicin | Adriamycin® Farmitalia | 0.5 μg ml$^{-1}$ | SW |
| Daunorubicin | Cerubidin®, Rohne-P | 0.1 μg ml$^{-1}$ | SW/PBS |
| VP-16 | Vepeside®, Bristol-Myers | 5 μg ml$^{-1}$ | PBS |
| Vincristine | Oncovin®, Lilly | 0.1 μg ml$^{-1}$ | PBS |
| Melphalan | Alkeran®, Wellcome | 2.5 μg ml$^{-1}$ | acid etOH |
| Prednisolon | Precortalon®, Organon | 10 μg ml$^{-1}$ | PBS |
| Mitoxantrone | Novantrone®, Lederle | 0.5 μg ml$^{-1}$ | PBS |
| 6-TG | Sigma | 10 μg ml$^{-1}$ | NaOH/SW |
| Amsa | Amsakrin®, Bristol-Myers | 0.5 μg ml$^{-1}$ | SW |

[a]EDCC; Empirically derived cut-off concentrations for *in vitro-in vivo* comparison were established as described previously (Larsson *et al.*, 1992*a*). SW = sterile water, etOH = absolute ethanol.

**Table II** Combinations tested

| *Combinations*[a] | *n* |
|---|---|
| A + Dnr (AraC + Daunorubicin) | 44 |
| A + Am (AraC + Amsa) | 44 |
| A + Mit (AraC + Mitoxantrone) | 44 |
| DAT (AraC + Doxorubicin + 6-thioguanine) | 42 |
| APV (AraC + Vincristine + Prednisolon) | 42 |
| MEA (AraC + VP16 + Mitoxantrone) | 37 |
| AVAm (AraC + VP16 + Amsa) | 37 |
| AraC + VP16 | 14 |
| VP16 + Mit | 14 |
| Amsa + Mit | 14 |
| VP16 + Amsa | 14 |

[a]Concentrations for the individual components used are EDCC listed in Table I.

### *FMCA procedure*

The principal steps of the assay procedure have been described previously (Larsson *et al.*, 1992*a*; Larsson & Nygren, 1990). Day 1 180 μl of the leukaemia preparation at 2.5–5 × $10^5$ cells ml$^{-1}$ in culture medium were seeded into the wells of V-shaped 96 well experimental microtiter plates (Nunc, Roskilde, Denmark) prepared as described above. Six blank wells received only culture medium and six wells with cells, but without drugs served as control. The culture plates were then incubated at 37°C in humidified atmosphere containing 95% air and 5% $CO_2$ for 72 h. At the end of the incubation period the plates were centrifuged (200 *g*, 7 min) and the medium removed by flicking the plate. After one wash with PBS, 200 μl of PBS containing FDA (10 μl ml$^{-1}$) was added columnwise to control, experimental and blank wells. Subsequently the plates were incubated for 1 h before reading the fluorescence in a Fluoroscan 2. The fluorometer was blanked against wells containing PBS including the fluorescent dye but without cells. The results obtained by the indicator FDA are presented as survival index (SI) defined as fluorescence in per cent of control cultures (Indicator test/Indicator control, with blank values subtracted). Quality criteria for a successful assay included an FDA signal in control cultures of $>5\times$ mean blank values, mean CV in control cultures of $<30\%$ and $>80\%$ of leukaemic cells prior to incubation.

### *Models of drug interactions* in vitro

Two models of combination chemotherapy were tested essentially according to the 'multiplicative' concept of drug interactions (Valeriote & Lin, 1975) and to the procedure and terminology used previously by Sondak *et al.* for *in vitro* testing of solid tumours (Sondak *et al.*, 1988*a*). The observed SI values of the combination were plotted against that predicted by two models. In the first model, termed 'additive', the effect of the combination is expected to be equal to the product of the effect of its constituents. Thus, a two-drug combination composed of single agents with SI values of 50 and 40%, the combination would be expected to result in a SI value of 20% ($0.5 \times 0.4 = 0.2$). In the second model, termed 'Dmax', the combination is expected to produce no greater effect than the most active single agent (Dmax) alone, thus the effect of the combination = Dmax. Observed values for combinations falling between these two models would represent sub-additive effects whereas those falling above Dmax would indicate true antagonism. Synergy was defined as values falling below those predicted by the additive model (Valeriote & Lin, 1975). In some analysis of the data, the ratio of observed SI values and those expected according to the additive model was plotted and observed/expected ratios $1 \pm 0.2$ were arbitrarily defined as additive interactions (Lepri *et al.*, 1991).

### *Statistical analysis*

The two models were compared using the non-parametric sign test. For each sample tested, the absolute value ($SI_a$) of ($/SI_a/$) the difference between the observed effect of the drug combination ($SI_{obs}$) and that predicted by each model ($SI_{pred}$) was calculated: $SI_{pred} - SI_{obs} = /SI_a/$. The value obtained for the Dmax model ($/SI_aDmax/$) was then subtracted from the value obtained for the additive model; ($/SI_aadd/$): $/SI_aadd/ - /SI_aDmax/$. A negative value would consequently indicate that the additive model is more accurate in predicting the observed result. The sign test was then used to determine if one model was significantly more likely to predict the observed result after excluding ties. The level of significance was set to $P<0.05$.

### In vitro-in vivo *comparison*

Patients were treated according to local protocols without knowledge of assay results. *In vivo* response was defined as complete response (CR) as previously described (Larsson *et al.*, 1992*a*). The patients included for correlation were those receiving relevant combination therapy with curative intent and in which a clear documentation of clinical response were available. The single agent *in vitro-in vivo* comparisons were based on the sensitivity of the most active single agent actually given *in vivo*. Correlations were performed at a previously specified concentration producing a significant scatter of SI values (Larsson *et al.*, 1992*a*) using either a fixed cut-off line (SI = 40%) determined from comparison with previous results using the differential staining cytotoxicity assay (Larsson *et al.*, 1992*b*) or the use of drug specific relative cut-off lines (median and median + 1 standard deviation (s.d.; Kern & Weisenthal, 1990; Bosanquet, 1991). Using the latter approach the SI values were positioned in relationship to the sensitivity of all other samples in the present study where low drug resistance (LDR) denotes SI values <median, intermediate drug resistance (IDR) values >

median but <1 s.d. and extreme drug resistance (EDR) which denotes SI values>median + 1 s.d. (Kern & Weisenthal, 1990; Bosanquet, 1991).

### Results

### *Effect of drug combinations*

In Figure 1 the percentile distribution of SI values is shown for each drug (a) and the combinations investigated (b). Each bar encompasses 90% of the observations. The median is indicated by the solid line whereas the broken lines delimits 25% of the observations. The median value is lower for all combinations compared to the most active single component.

### *Type of interaction for different combinations*

In Figure 2 the expected SI values are plotted against those observed for the AraC + Amsa and APV combinations. A large variability is not only noted between individual samples (Figure 2) but also observed for individual samples in response to different combinations (Figure 3). For AraC + Amsa the additive model provides a better distribution of points about the line of identity whereas the opposite is true for the APV combination. When the models were statistically compared using the sign test, the additive model provided a preferable fit for AraC + Amsa, MEA and AraC + Dnr ($P<0.05$; Table III). The Dmax model was superior only in the case of APV ($P<0.05$) whereas no preference for either model was evident for the remaining combinations (non-significant; Table III). In an attempt to investigate which of the individual components that contributed most to the efficacy of MEA, a second series of experiments ($n$ = 14) was performed with seven two-drug combinations (Figure 3). The results show that VP16 + Mit was the far most active two-drug combination in terms of additive and synergistic interactions (Figure 3). In eight out of 14 samples (57%) the interaction was synergistic compared to two of 14 (14%) and four of 14 (29%) for AraC + VP16 and AraC + Mit, respectively (Figure 3).

### *Use of Dmax for prediction of combination activity*

The accuracy of the most effective single agent in predicting the *in vitro* activity of the combination using an SI value of <40% as for separation of sensitive from resistant is shown in Table IV. Of the 290 tests on combinations, 248 (85%) showed an agreement between *in vitro* results of the best single agent and the combination. The false negative rate was 12%. It should be noted that in 61% of the false negatives, the SI value of the most active single agent were >40 but <50% (not shown).

### *Relationship to clinical outcome*

When the two interaction models were related to treatment outcome ($n$ = 23) three out of eight (38%) of the non-responders showed preference for the additive model whereas the corresponding figure for patients responding to given combination therapy was 12 out of 15 (80%; Table V). Direct comparison of Dmax and the combination for correlation with clinical outcome using a 40% cut-off line revealed a false positive (SR) rate of 25 and 38% for Dmax and the combination, respectively (Table V). The corresponding values for the false negative (RS) rate was 20 and 13%. This pattern was reversed when drug-specific cut-off lines (median value) was used for separation of sensitivity and resistance. The SR and RS rate was in this case 38 and 13 *vs* 25 and 20% for Dmax and the combination, respectively (Figure 4). The total number of patients given a correct classification in terms of true positives (SS), true negatives (RR), SR and RS was 18/23 (78%) irrespective of cut-off lines employed or whether Dmax or observed combination activity provided the basis for the correlations (Figure 4, Table V).

### Discussion

The additive model was statistically preferable in three out of seven combinations tested by the sign test, including the Dnr + AraC, which is empirically known to be one of the clinically most active remission induction regimens (Marie & Zittoun, 1991). For the APV combination, on the other hand, the Dmax model more closely predicted the effect. This is not surprising since Vcr and Pred has shown to be of little value for remission induction in AML (Goldman & James, 1990) and is only marginally active *in vitro* at concentrations which are clearly active in ALL (Larsson *et al.*, 1992*a*). Furthermore, when 6TG was added to an anthracycline + AraC containing regimen (DAT), no preference between the models was evident. This is compatible with the observation that no difference in terms of clinical CR rate at remission induction between AraC + anthracycline alone and the use of the same

**Figure 1** Effect of individual antileukaemic drugs (**a**) and their combinations (**b**, $n$ = 36–44) at EDCC on SI in AML samples. Each bar encompasses 90%. The solid line within the bar indicate the median. The two broken lines delimits 25% of all the observed SI values.

**Figure 2** Relationship between expected SI values (x-axis) according to the additive (right) and Dmax model (left) and those observed experimentally (y-axis) for AraC + Amsa (upper) and APV (lower). The solid line is the line of identity.

**Table III** Comparison between Dmax and the additive model for drug interactions

| *Combinations* | Sign – | Sign + | – % | *Probability* P |
|---|---|---|---|---|
| A + Dnr | 25 | 14 | 64 | <0.05 |
| A + Am | 28 | 14 | 67 | <0.05 |
| A + Mit | 17 | 25 | 40 | n.s. |
| DAT | 22 | 18 | 55 | n.s. |
| APV | 13 | 29 | 31 | <0.05 |
| MEA | 24 | 12 | 67 | <0.05 |
| AVAm | 21 | 15 | 58 | n.s. |

Statistical analysis performed using the sign test, see Materials and Methods. n.s. = non-significant.

regimen in conjunction with 6TG has been established (Marie & Zittoun, 1991). When the relationship to clinical outcome was investigated it became apparent that patients responding to therapy showed a higher preference for the additive model compared to Dmax whereas the opposite was true for patients not responding to therapy. The fact that non-responders showed preference for the Dmax model is in accordance with the study of Sondak in which the majority of samples were from drug resistant tumours where activity and interactions of multiple drugs are expected to be at a minimum. The responding patient population, on the other hand, is drug sensitive and the probability for true drug interactions is consequently expected to be higher. However, one should also note that the frequency of additive interactions in the present study may be underestimated in comparison with the report of Sondak *et al.* due to the different endpoints used (cell viability *vs* cell proliferation).

One especially interesting observation concerns the fact that the addition of VP16 to the AraC + Mit combination makes this combination more predictable by the additive compared to the Dmax model. This potential drug interaction has been exploited clinically in Mit + VP16 + AraC containing regimens with good clinical CR rate also in relapsed and refractory patients (Amadori *et al.*, 1991; Björkholm *et al.*, 1990; Link *et al.*, 1990). Furthermore, we have recently shown that AraC, Mit and VP16 appears to be mutually non-cross resistant *in vitro* in AML samples (Kristensen *et al.*, 1992) which may indicate additional mechanisms of resistance other than the 'classical' multidrug resistance phenotype or alterations of topoisomerase 2. The important interaction may be between VP16 and Mit, since the addition of VP16 to an Amsa + AraC combination shifts the preference of the additive model to non-significance. Furthermore, the direct testing of VP16 + Mit showed the highest frequency of synergistic interactions among all tested two-drug combinations, including Arac + Mit and AraC + VP16. Good results in refractory leukaemia has been obtained with the VP16 + Mit combination (Ho *et al.*, 1988; Lazzarino *et al.*, 1989). This observation may become of clinical importance and indicate the feasibility of FMCA to detect drug interactions in fresh tumour cell samples from patients with leukaemia.

However, extrapolation of *in vitro* results have to be done with care since important differences exist between *in vitro* systems and the *in vivo* situation with respect to drug interactions. First, it is important to note that even subadditive effects at the cellular level may translate into therapeutically beneficial interactions *in vivo* (Valeriote & Lin, 1975). Second,

**Figure 3** Drug interactions in 14 different samples exposed to seven two-drug combinations. The ratio of observed SI values and those calculated using the additive model is plotted for each sample. Additive interactions was arbitrarily defined as observed/expected ratios ranging between 1 ± 0.2. Subadditive values were consequently those above 1.2 and synergistic values those falling below 0.8 as indicated. The individual patients are connected with a solid line to ease identification.

**Table IV** Correlation between the activity of the most active agent (Dmax) and the combination

| *Dmax/comb* | *No. of samples* | *%* |
|---|---|---|
| Sensitive/Sensitive | 182 | 62 |
| Sensitive/Resistant | 8 | 3 |
| Resistant/Sensitive | 34 | 12 |
| Resistant/Resistant | 66 | 23 |
| Totals | 290 | 100 |

An SI value of 40% was used for separation of sensitive samples from resistant ones.

tumour cells are exposed to high drug concentrations *in vitro* (in most cases equivalent to peak plasma levels) which gives much higher concentration × time products (CXT) than obtained *in vivo*. However, this may not be the case for all drugs. For example, bioassay determinations of Dox have shown similar half lifes *in vitro* and *in vivo* (Hildebrandt-Zanki & Kern, 1986). For the majority of antineoplastic drugs, the *in vitro* assay CXT:s of active drug have not been determined. Third, each drug was tested at a single concentration only, due to limited availability of cells. Optimally, testing a range of concentrations allowing formal isobologram analysis to be performed would yield more information on subtle drug interactions (Berenbaum, 1989). Fourth, no attempt was made to reproduce the scheduling employed in various drug combinations. However, the significance of scheduling and cell cycle specific interactions has not been established for most clinically employed drug combinations and the relevance of these phenomena to the current study remains uncertain.

Single agents were also tested for their ability to predict the effect of a combination. This has important implications for drug sensitivity testing using *in vitro* assays since in most cases drug combinations are not tested. Drug interactions adhering to the additive model would in this case be expected to cause false-negative *in vitro/in vivo* correlations. In the majority of cases the most active single agent correctly predicted the *in vitro* activity of the combination, based on a 40% SI cut-off limit for discriminating sensitive from resistant (Larsson *et al.*, 1992*b*). Thus, in 15% of the cases the

**Table V** Relationship of the interaction models to clinical outcome[a]

| *Pat no.* | *Treatment* | *Result* | *Best model* | *Correlation Dmax* | *Correlation Combination* |
|---|---|---|---|---|---|
| 1 | AraC + Dnr | NR | Dmax | RR | RR |
| 2 | AraC + Dnr | NR | Additive | RR | SR |
| 3 | AraC + Mit | NR | Dmax | RR | RR |
| 4 | AraC + Dnr | NR | Additive | RR | RR |
| 5 | DAT + Vcr | NR | Additive | SR | SR |
| 6 | AraC + Dnr | NR | Dmax | RR | RR |
| 7 | AraC + Mit | NR | Dmax | SR | SR |
| 8 | AraC + Dnr | NR | Dmax | RR | RR |
| 9 | AraC + Amsa | CR | Additive | SS | SS |
| 10 | DAT + Vcr | CR | Dmax | SS | SS |
| 11 | AraC + Dnr | CR | Dmax | SS | SS |
| 12 | MEA | CR | Additive | SS | SS |
| 13 | AraC + Dnr | CR | Dmax | SS | SS |
| 14 | MEA | CR | Additive | SS | SS |
| 15 | AraC + Dnr | CR | Additive | SS | SS |
| 16 | MEA | CR | Additive | SS | SS |
| 17 | AraC + Dnr | CR | Additive | SS | SS |
| 18 | AraC + Dnr | CR | Additive | RS | RS |
| 19 | AVAm | CR | Additive | RS | RS |
| 20 | AraC + Dnr | CR | Additive | RS | SS |
| 21 | AraC + Dnr | CR | Additive | SS | SS |
| 22 | AraC + Dnr | CR | Additive | SS | SS |
| 23 | AraC + Dnr | CR | Additive | SS | SS |

[a]Patients listed are those treated with combination therapy with curative intent and where clinical data was evaluable for correlation. NR = Nonresponders and CR = complete response according to the definitions in Materials and methods. Best model refers to the interaction model which was most accurate in predicting the effect of the combination as described in Materials and methods. SS = true positives, SR = false positives, RR = true negatives, RS = false negatives. A 40% cut-off limit was used for separation of *in vitro* sensitivity and resistance. For patient 5 and 10 who received Vcr in addition to DAT, the latter combination (DAT) was used for the *in vitro* predictions.

**Figure 4** Dmax (●) or combination activity (○) as the basis for correlation to clinical outcome ($n = 23$) using drug specific relative cut-off lines. The median value separates LDR from IDR and median + 1 s.d. separates IDR from EDR (Weisenthal & Kern, 1990; Bosanquet, 1991). See Materials and methods. Overall response rate was 65%.

activity of the most active single agent failed to predict the combination. The testing of combinations may thus be required in order to achieve maximal predictive accuracy. However, comparison of direct clinical correlations based on combinations *vs* single agents showed no apparent advantage using the former approach. Although the tendency to produce false positive over false negative results varied in opposite directions for correlation procedures based on Dmax *vs* combination activity depending on which type of cut-off limit was used, the overall predictive ability was similar. Using the relative cut-off lines, the same EDR cases could be identified with both methods. It should be noted that 62% of the false negatives had SI values for most active single agent activity of $<50\%$ suggesting that effective combinations requires active components. Additive and synergistic interactions may thus be more important for predictions of cure and survival than for predicting CR. This possibility is currently investigated.

This study was supported by grants from the Swedish Cancer Society (2695-B89-OIX).

### References

AMADORI, S., ARCESE, W., ISACCI, G., MELONI, G., PETTI, M., MONARCA, B., TESTI, A.M. & MANDELLI, F. (1991). Mitoxantrone, etoposide, and intermediate-dose cytarabine: an effective and tolerable regimen for the treatment of refractory acute myeloid leukemia. *J. Clin. Oncol.*, **9**, 1210–1214.

BERENBAUM, M.C. (1989). What is synergy. *Pharmacol. Rev.*, **41**, 93–132.

BJÖRKHOLM, M., BJÖRNSDOTTIR, J., STENKE, L. & GRIMFORS, G. (1990). Mitoxantrone, etoposide and cytarabine in the treatment of acute non-lymphocytic leukemia. *Oncology*, **47**, 112–114.

BOSANQUET, A. (1991). Correlations between therapeutic response of leukemias and *in vitro* drug sensitivity assay. *Lancet*, **1**, 711–714.

GOLDMAN, J. & JAMES, N. (1990). Leukemia and bone marrow transplantation. In *Treatment of Cancer*, Sikora, K. & Halnan, K.E. (eds) pp. 679–695. Chapman and Hall: London.

HILDEBRANDT-ZANKI, S.U. & KERN, D.H. (1986). A rapid bioassay to determine stabilites of anticancer agents under conditions of the clonogenic assay. *In Vitro Cell Dev.*, **22**, 247–252.

HO, A., LIPP, T., EHNINGER, G., ILLIGER, H., MEYER, P., FREUND, M. & HUNSTEIN, W. (1988). Combination of mitoxantrone and etoposide in refractory acute myelogenous leukemia: an effective and well-tolerated regimen. *J. Clin. Oncol.*, **6**, 213–217.

HONGO, T., FUJII, Y. & IGARASHI, Y. (1990). An *in vitro* chemosensitivity test for the screening of anti-cancer drugs in childhood leukemia. *Cancer*, **65**, 1263–1272.

KERN, D. & WEISENTHAL, L. (1990). Highly specific prediction of antineoplastic drug resistance with an *in vitro* assay using supra-pharmacologic drug exposures. *J. Natl Cancer Inst.*, **82**, 582–588.

KRISTENSEN, J., JONSSON, B., SUNDSTRÖM, C., NYGREN, P. & LARSSON, R. (1992). *In vitro* analysis of drug resistance in tumor cells from patients with acute myelocytic leukemia. *Med. Oncol. Tumor Pharmacother.*, **9**, 65–73.

LARSSON, R., KRISTENSEN, J., SANDBERG, C. & NYGREN, P. (1992*a*). Laboratory determination of chemotherapeutic drug resistance in tumor cells from patients with leukemia using a fluorometric microculture cytotoxicity assay (FMCA). *Int. J. Cancer*, **50**, 177–185.

LARSSON, R., JONSSON, B., KRISTENSEN, J., ÖBERG, G., SIMONSSON, B., SUNDSTRÖM, C., LÖNNERHOLM, G., KREUGER, A., GLIMELIUS, B., HAGBERG, H. & NYGREN, P. (1992*b*). Drug sensitivity testing of tumor cells from patients with acute leukemia and non-Hodgkin's lymphoma using a fluorometric microculture cytotoxicity assay. International symposium on the clinical value of drug resistance assays in leukemia and lymphomas, Amsterdam, March 16, P46 (Meeting abstract).

LARSSON, R. & NYGREN, P. (1990). Pharmacological modification of multi-drug resistance (MDR) *in vitro* detected by a novel fluorometric microculture cytotoxicity assay. Reversal of resistance and selective cytotoxic actions of cyclosporin A and verapamil on MDR leukemia T-cells. *Int. J. Cancer*, **46**, 67–72.

LAZZARINO, M., MORRA, E., ALESSANDRINO, E., ORLANDI, E., PAGNUCCO, G., MERANTE, S., BERNASCONI, P., INVERARDI, D., BONFICHI, M. & BERNASCONI, C. (1989). Mitoxantrone and etoposide: an effective regimen for refractory or relapsed acute myelogenous leukemia. *Eur. J. Hematol.*, **43**, 411–416.

LEPRI, E., BARZI, A., MENCONI, E., PORTUESI, M.G. & LIBERATI, M. (1991). *In vitro* synergistic activity of PDN-IFNa and NM + IFNa combinations on fresh bone-marrow samples from multiple myeloma patients. *Hematol. Oncol.*, **9**, 79–86.

LINK, H., FREUND, M., DIEDRICH, H., WILKE, H., AUSTEIN, J., HENKE, M., WANDT, H., FACKLER-SCWALBE, E., SCHLOMOK, G. & HOFFMAN, R. (1990). Mitoxantrone, cytosine arabinoside, and VP-16 in 36 patients with relapsed and refractory acute myeloid leukemia. *Haematol. Bluttransfus.*, **33**, 322–325.

MARIE, J. & ZITTOUN, R. (1991). Chemotherapy of acute myelogenous leukemia. *Bailliere's Clinical Hematol.*, **4**, 97–110.

PIETERS, R., HUISMANS, D., LOONEN, A.H., HÄHLEN, K., VAN DER DOES-VAN DEN BERG, A., VAN WERING, E.R. & VEERMAN, A.J.P. (1991). Relation of cellular drug resistance to long-term clinical outcome in childhood acute lymphoblastic leukemia. *Lancet*, **338**, 399–403.

RANKIN, E.M. & KAYE, S.B. (1990). Principles of chemotherapy. In *Treatment of Cancer*, Sikora, K. & Halnan, K.E. (eds) pp. 127–145. Chapman and Hall: London.

SONDAK, V., KORN, E. & KERN, D. (1988*a*). *In vitro* testing of chemotherapeutic combinations in a rapid thymidine incorporation assay. *Int. J. Cell Cloning*, **6**, 378–391.

SONDAK, V., KORN, E., MORTON, D. & KERN, D. (1988*b*). Testing chemotherapeutic combinations in the human tumor colony-forming assay. *J. Surg. Oncol.*, **37**, 156–160.

VALERIOTE, F. & LIN, H. (1975). Synergistic interaction of anticancer agents: a cellular perspective. *Cancer Chemother. Rep.*, **59**, 895–900.

VON HOFF, D. (1988). Human tumor cloning assays: applications in clinical oncology and new antineoplastic agent development. *Cancer Met. Rev.*, **7**, 357–371.

WEISENTHAL, L. & LIPPMAN, M. (1985). Clonogenic and non-clonogenic *in vitro* chemosensitivity assays. *Cancer Treat. Rep.*, **69**, 615–632.